# Cytosolic DNA sensors and glial responses to endogenous DNA

**DOI:** 10.3389/fimmu.2023.1130172

**Published:** 2023-03-14

**Authors:** Alexander J. Suptela, Ian Marriott

**Affiliations:** Department of Biological Sciences, University of North Carolina at Charlotte, Charlotte, NC, United States

**Keywords:** genomic integrity, DNA sensors, neuroinflammation, neurodegeneration, glia

## Abstract

Genomic instability is a key driving force for the development and progression of many neurodegenerative diseases and central nervous system (CNS) cancers. The initiation of DNA damage responses is a critical step in maintaining genomic integrity and preventing such diseases. However, the absence of these responses or their inability to repair genomic or mitochondrial DNA damage resulting from insults, including ionizing radiation or oxidative stress, can lead to an accumulation of self-DNA in the cytoplasm. Resident CNS cells, such as astrocytes and microglia, are known to produce critical immune mediators following CNS infection due to the recognition of pathogen and damage-associated molecular patterns by specialized pattern recognition receptors (PRRs). Recently, multiple intracellular PRRs, including cyclic GMP-AMP synthase, interferon gamma-inducible 16, absent in melanoma 2, and Z-DNA binding protein, have been identified as cytosolic DNA sensors and to play critical roles in glial immune responses to infectious agents. Intriguingly, these nucleic acid sensors have recently been shown to recognize endogenous DNA and trigger immune responses in peripheral cell types. In the present review, we discuss the available evidence that cytosolic DNA sensors are expressed by resident CNS cells and can mediate their responses to the presence of self-DNA. Furthermore, we discuss the potential for glial DNA sensor-mediated responses to provide protection against tumorigenesis versus the initiation of potentially detrimental neuroinflammation that could initiate or foster the development of neurodegenerative disorders. Determining the mechanisms that underlie the detection of cytosolic DNA by glia and the relative role of each pathway in the context of specific CNS disorders and their stages may prove pivotal in our understanding of the pathogenesis of such conditions and might be leveraged to develop new treatment modalities.

## 1 Introduction

In 2022, there were an estimated 1.9 million new cancer cases and over 600,000 deaths due to these diseases in the United States (1). Of these, approximately 25,000 were associated with the brain and the central nervous system (CNS), and these have a poor prognosis with only a 5-year survival rate of 32.5% (2). Additionally, lethal cases of neurodegenerative diseases including Alzheimer’s disease (AD) and Parkinson’s disease (PD) are becoming increasingly prevalent, and this is a serious concern as an estimated 6.5 million Americans were reported to live with AD in 2022 and 1 million having PD in 2019 (3, 4). While multiple mechanisms, including cellular senescence and dysfunctional mitophagy, have been associated with cancer development and age-related diseases (5–8), genomic instability arising from excessive DNA damage and/or DNA repair mechanism dysfunction is recognized as a key driving force for such diseases in general (9), and the development of CNS cancers and neurodegenerative diseases more specifically (10, 11).

High fidelity DNA repair is required to maintain genomic integrity. Breaks in DNA are a relatively common occurrence and can occur naturally up to tens of thousands of times per day, per cell. However, the number of such breaks can be drastically increased by exogenous insults such as ionizing radiation (IR) and oxidative stress. Exposure to high levels of IR can be tremendously detrimental and affects nearly every macromolecule in a cell. These effects can either occur directly or indirectly. For example, IR alone directly disrupts the structure of DNA, creating breaks in its molecular backbone (12). Alternatively, IR can elicit radiolysis of cellular water whereby the chemical bonds of water molecules are broken down resulting in the generation of reactive oxygen species (ROS) including hydroxyl radicals, ionized water, superoxide anions, and hydrogen peroxide (12). While ROS play essential roles in many cellular processes, excessive levels of ROS disrupt redox homeostasis and can induce DNA lesions (13). DNA lesions/breaks initiate DNA damage responses (DDR), a collection of mechanisms that ensure efficient DNA repair and maintain genomic integrity (14, 15). However, DNA repair is not infallible. If cells are permitted to replicate with unrepaired or incompletely repaired DNA, an accumulation of mutations and DNA damage may lead to cancer development, neurodegenerative disorders, and other age-related diseases. Furthermore, DNA damage has recently been implicated in the generation of detrimental inflammation, and this effect is often associated with the presence of self-DNA in the cytoplasm (16–19).

DNA damage due to exposure to IR, oxidative stress, or even chemotherapy, results in cytosolic DNA accumulation, often in the form of micronuclei (20). Micronuclei are small nuclei-like structures containing lagging or damaged chromosome fragments that continue into the interphase following completion of mitosis or meiosis (20) The nuclear envelopes surrounding micronuclei are typically defective and prone to rupture, after which, their DNA cargo is liberated into the cytosol. Additionally, micronuclei have been described as a source of complex genome rearrangements, including one-off catastrophic rearrangement events known as chromothripsis (21). Furthermore, the presence of micronuclei is associated with many autoimmune diseases (22–26), neurodegenerative diseases (27–32), and aggressive cancers (33) in affected tissues. Indeed, the presence of micronuclei has historically been used as a means to assess the genotoxicity of chemicals and mutagens *via* the cytokinesis-block micronucleus assay, and their contribution to the initiation of innate immune responses is now becoming apparent (34).

While micronuclei are likely to be a major source of cytoplasmic genomic self-DNA, mitochondria may also serve as a source of DNA in the cytosol (35). Mitochondria primarily function to produce the ATP necessary for normal cell activity *via* oxidative phosphorylation. Outside of oxidative phosphorylation, mitochondria also perform many other metabolic and non-metabolic roles ranging from the regulation of apoptosis to the generation of ROS that are necessary for maintaining redox homeostasis (36, 37). What makes mitochondria truly unique, however, is that they contain their own circular genome due to their endosymbiotic origin (38). The various means by which mitochondrial contents, including mitochondrial DNA (mtDNA), are released have been extensively reviewed elsewhere (39–44), but generally involves passive/accidental release due to mitochondrial stress and dysfunction, and cell death pathways including necrosis and apoptosis (40).

Since DNA is normally sequestered in the nucleus and mitochondria, its presence in the cytosol can function as a damage associated molecular pattern (DAMP) and serve to trigger inflammatory innate immune responses. In the CNS, such responses to both endogenous and exogenous insults must be tightly regulated to avoid damaging, or even lethal, inflammation. While the recruitment of peripheral leukocytes to the CNS and their subsequent activation are important in the development of immune responses in disease states, it is now recognized that resident glial cells, such as microglia and astrocytes, play a critical role in the initiation of detrimental neuroinflammation.

## 2 Glial cells play a critical role in the initiation and progression of immune responses in the CNS

In the healthy brain, microglia and astrocytes are essential for homeostasis. Microglia perform critical housekeeping functions such as synaptic remodeling and pruning (45, 46), and removal of cellular debris and dead or dying cells (47), which is necessary for creating a regenerative environment (48). Astrocytes are the most abundant glial cell in the brain and have a crucial role in synaptogenesis, synaptic transmission, and neurotransmitter recycling, and for the maintenance of the blood brain barrier (BBB) (49–53). Importantly, it is now apparent that microglia and astrocytes both serve as sentinel cells that initiate and exacerbate immune responses associated with CNS pathology (54, 55). Given their wide distribution throughout the CNS, they are ideally positioned to confront and respond to trauma or invading pathogens. In disease states, microglia and astrocytes are activated and produce a wide array of potent proinflammatory mediators, such as IL-6, IL-1β, and TNF, as well as chemokines that promote the recruitment of peripheral leukocytes across the BBB that can further contribute to potentially damaging neuroinflammation (56–59). The initiation of microglial and astrocytic responses is now recognized to be mediated by multiple families of cell surface, endosomal, and cytosolic PRRs that are triggered by DAMPs and pathogen-associated molecular patterns (PAMPs). This subsequently results in the activation of transcription factors that precipitate the production of cell surface and secreted immune mediators.

Of these PRRs, perhaps the best studied are the cell surface and endosomal Toll-like receptors (TLRs) and the cytosolic nucleotide-binding and oligomerization domain-containing (NOD)-like receptors (NLRs), and these sensors have been exhaustively studied for their roles in antimicrobial and antiviral responses (as reviewed in 60–69). The TLR and NLR families consist of at least 10 and 22 members in mammals, respectively, and these receptors are widely expressed throughout the body on/in peripheral leukocytes and non-leukocytic cell types (70–72). Importantly, we, and others, have demonstrated the constitutive and/or inducible expression of TLRs (54, 55, 58, 73–75) and the NLRs, NOD1 and NOD2 (76–78), on/in both microglia and astrocytes.

These TLRs and NLRs can detect a variety of bacterial or viral extra- and intracellular PAMPs and DAMPs to activate downstream signaling cascades and initiate proinflammatory and/or antiviral activity by glial cells. However, NOD1/2 appears to be limited to the detection of bacterial cell wall components (78). Furthermore, while several TLRs are able to detect and respond to nucleic acids in endosomal compartments (72, 79, 80), they are not well suited to detect compromised cytosolic sterility or damage. Rather, it now appears that cells utilize discrete cytosolic PRR families that are capable of responding to the presence of foreign and/or self-nucleic acids in the cytoplasm.

## 3 Detection of cytosolic nucleic acids by glial cells

PRR families, in addition to TLRs and NLRs, have recently been identified that serve as cytosolic sensors for foreign or altered self-nucleic acids (81). These include the retinoic acid-inducible gene I (RIG-I)-like family of receptors (RLRs) that can detect bacterial and viral nucleic acids and, more specifically, dsRNA (82–84). There are currently three known RLRs: RIG-I, melanoma differentiation-associated gene 5 (MDA5), and laboratory of genetics and physiology 2 (LGP2). All three RLRs share a common central helicase domain and carboxy-terminal domain, although only RIG-I and MDA5 possess two caspase activation and recruitment domains (CARDs) that are required for downstream signal transduction (85). In contrast, LGP2 lacks a CARD domain and, instead, negatively regulates RIG-I and MDA5 activity (85, 86). Upon RNA binding, RIG-I and MDA5 interact with the CARD domain found on mitochondrial antiviral-signaling (MAVS), a mitochondria-localized adaptor protein, which subsequently activates TRAF family member-associated nuclear factor-kappa-B activator (TANK)-binding kinase 1 (TBK1) and inhibitor of nuclear factor kappa-B kinase subunit epsilon (IKKϵ). TBK1 and IKKϵ, in turn, activate the transcription factors interferon regulatory factors 3 and 7 (IRF3 and IRF7), which finally induce the transcription of type-I interferons (IFNs) among other antiviral mediators. Importantly, we have shown that RIG-I and MDA5 are constitutively expressed in microglia and astrocytes and that such expression is upregulated following viral infection (87). Furthermore, we have recently demonstrated that RIG-I not only recognizes viral dsRNA in glia, but is also able to respond to bacterial dsRNA (88).

Surprisingly, RIG-I may elicit immune responses to cytosolic DNA in addition to RNA, albeit in an indirect manner mediated by the actions of RNA polymerase III (RP3) (88–93), wherein RP3 reverse transcribes cytosolic dsDNA into a 5’ triphosphate-containing dsRNA ligand that then can subsequently be detected by RIG-I. Such a mechanism might explain the earlier, and perhaps erroneous, description of RP3 as a DNA sensor (92, 93).

However, it is now apparent that cells, including glia, possess a number of molecules that specifically serve as cytosolic DNA sensing molecules to initiate responses to foreign or self-DNA. The first of such molecules to be discovered was DNA sensor Z-DNA binding protein (ZBP1; previously known as DNA-dependent activator of IRFs (DAI)) in 2007 (94). Since then, our knowledge of cytosolic DNA sensing PRRs has expanded to include proteins such as cyclic GMP-AMP synthase (cGAS) (95), interferon gamma-inducible 16 (IFI16) (96), and absent in melanoma 2 (AIM2) (97–99). Importantly, we, and others, have described the expression and activity of cGAS (100, 101), IFI16 (102–104), ZBP1 (105–108), and AIM2 (101, 109, 110), in resident CNS cells including human and murine microglia and/or astrocytes.

**Figure 1:**
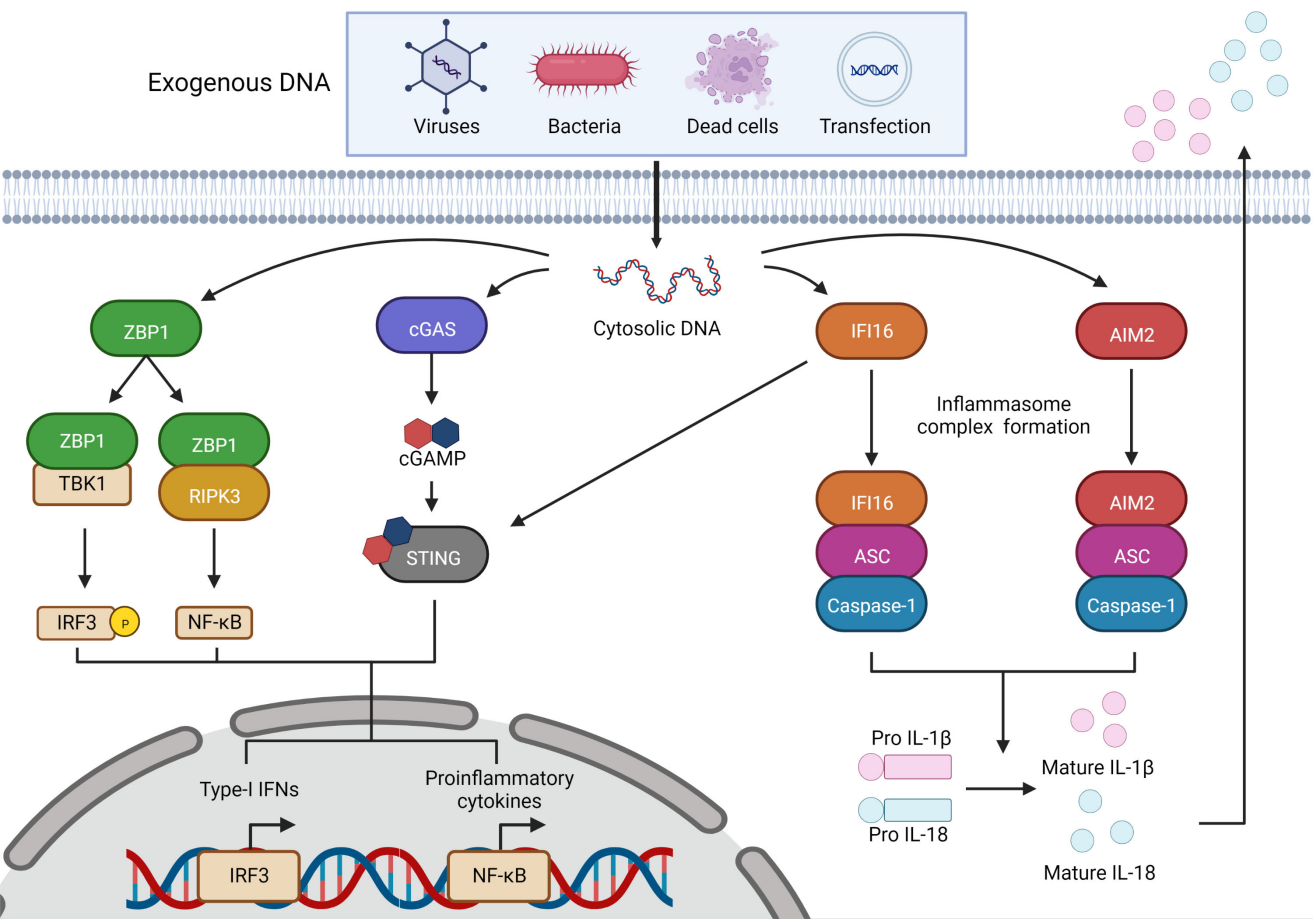
Cytosolic DNA sensors and their signaling pathways. Sensing of dsDNA by ZBP1 induces its association with RIPK3 and subsequent activation of NF-κB to elicit pro-inflammatory cytokine production, and/or its interaction with TBK1 to induce IRF3 activation and type-I IFN expression. Sensing of dsDNA by cGAS catalyzes the production of cGAMP that activates STING and leads to TBK1 activation that induces IRF3 activation and type-1 IFN expression. IFI16 can directly interact with STING following DNA sensing resulting in NF-κB and IRF3 activation, or can associate with ASC to form an inflammasome complex resulting in caspase-1-mediated IL-1β and IL-18 release. AIM2 sensing of dsDNA also leads to inflammasome complex formation with ASC and mature IL-1β and IL-18 release. This figure was created with Biorender.com.

As shown in **Figure 1**, cGAS and IFI16 use the endoplasmic reticulum-localized protein stimulator of interferon genes (STING) as an adaptor molecule to exert their responses, while ZBP1 and AIM2 recruit their own adaptor molecules, receptor interacting serine/threonine-protein (RIP) kinases 1 and 3 (RIPK1/3), and apoptosis-associated speck-like protein containing a CARD (ASC), respectively (99, 111). Upon activation, STING facilitates the activation of transcription factors TBK1, IRF3, and nuclear factor kappa-B (NF-κB) that then initiate the transcription of type-I IFNs, proinflammatory cytokines, and chemokines (112). ZBP1 recruits RIPK1 and 3 *via* interactions with its RIP homotypic interaction motif (RHIM) domains, which subsequently activates NF-κB (113). Both IFI16 and AIM2 can, like other receptors upstream of inflammasome formation, recruit ASC following DNA detection (99, 114). AIM2 and IFI16 can both form an inflammasome complex with ASC to recruit the effector protein, caspase-1, that subsequently cleaves pro-IL-1β and pro-IL18 to their mature forms for release (**Figure 1**) (99, 114). Additionally, AIM2-ASC inflammasome complex formation can lead to pyroptotic cell death *via* cleavage of gasdermin-D (GSDMD) (97, 98).

Finally, several other putative DNA sensors such as DNA-dependent protein kinase (DNA-PK) (115), DEAD-box helicase 41 (DDX41) (116), and meiotic recombination 11 homolog A (MRE11) (117) have been described in peripheral cell types, but their functions as PRRs for DNA in the CNS have not been investigated to date.

Peripheral and resident CNS cells exhibit inflammatory phenotypes following DNA damage (118–120), although the mechanisms underlying the initiation of these responses have remained elusive. Interestingly, several cytosolic DNA sensors appear to be capable of detecting mitochondrial and genomic self-DNA resulting from insults such as oxidative stress and IR (41, 121–128). As such, cytosolic DNA sensors may be the link between DNA damage and subsequent innate immune responses.

## 4 Cytosolic DNA sensors lie at the intersection of DNA damage and innate immunity

The presence of DNA in the cytoplasm is indicative of cell compromise as it is normally confined to the nucleus or mitochondria in healthy cells. While cytosolic DNA sensors have been extensively studied with regard to bacterial and viral infections, it has only been recently recognized that they may also play a critical role in the generation of immune responses to cytosolic mtDNA and genomic self-DNA (42, 121, 129–131). In contrast to endosomal nucleic acid sensors such as TLR9 that detect prokaryotic DNA based on their distinct methylation patterns (132), cytosolic DNA sensors seem to be unable to discriminate between foreign and self-DNA, and so can detect and respond to either (133, 134). However, it remains unclear whether such responses to self-DNA are protective or detrimental, especially in the context of the CNS where there is strong potential for damaging neuroinflammation. Regardless, it is now apparent that glial cells express multiple sensors capable of initiating their immune functions in response to the presence of cytosolic DNA as described below.

### 4.1 cGAS

Of all the DNA sensors, cGAS has risen to the forefront of nucleic acid sensor research since it’s discovery in 2013 (95). Over the past decade, our understanding of its role has expanded from the triggering of antiviral immunity to include the inhibition of homologous recombination-mediated DNA repair (135, 136), control of DNA replication dynamics (137), cellular senescence (35, 138–141), cell death (142–147), and tumorigenesis (136).

**Figure 2:**
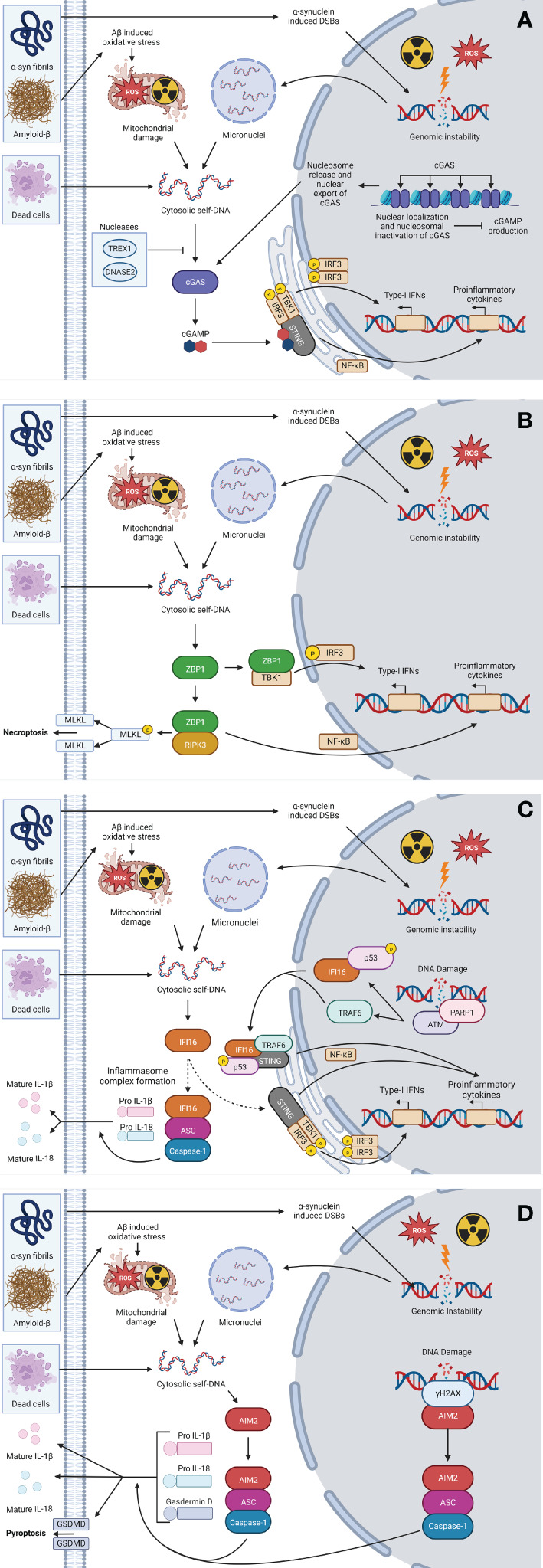
Cytosolic self-DNA sensing pathways. In the CNS, the uptake of α-Syn fibrils, amyloid-β, and/or cellular debris by resident CNS cells, or exposure to exogenous oxidative stress or ionizing radiation (IR), can lead to liberation of self-DNA to the cytosol *via* mitochondrial damage or genomic instability leading to micronuclei formation. The presence of self-DNA in the cytoplasm can then be perceived *via* DNA sensing PRRs including cGAS **(A)**, ZBP1 **(B)**, IFI16 **(C)**, and/or AIM2 **(D)**, leading to sensor-specific signaling pathways that precipitate the production of inflammatory cytokines, type-I IFNs and/or immunogenic necroptotic and pyroptotic cell death pathways. This figure was created with Biorender.com.

As shown in **Figure 2A**, following DNA binding, cGAS catalyzes the production of the secondary messenger molecule 2’3’cyclic guanosine monophosphate-adenosine monophosphate (cGAMP), which subsequently binds to STING that is located on the surface of the endoplasmic reticulum (95, 148, 149). Following interaction with cGAMP, STING undergoes a conformational change that initiates the recruitment of TBK1, which then phosphorylates IRF3 and liberates NF-κB. Activation of this signaling cascade results in the production of potent proinflammatory cytokines and chemokines including IL-6, TNF, and IL-8, and the type-I IFNs such as IFN-β (112, 150–152).

The discrimination, or lack thereof, between foreign and self-DNA has been recognized as a significant issue regarding cytosolic DNA sensors since indiscriminate DNA binding could lead to the development of autoimmune responses. Indeed, several autoimmune diseases, including Aicardi-Goutières syndrome (AGS), are associated with increased levels of cytoplasmic DNA and inflammatory mediator production (153). While DNA-mediated activation of cGAS occurs in a length dependent manner, with robust activity occurring only with DNA longer than 45 bp or shorter DNA fragments with flayed ends (154, 155), its ability to discriminate between DNA fragments seems to end here.

DNA damage and genomic instability has long been known to induce inflammatory responses (123, 156, 157), but it has only been recently that this phenomenon has been shown to be connected to cytosolic nucleic acid sensors including cGAS. In 2017, Mackenzie et al. (121) discovered that a primary culprit in the induction of inflammatory responses following IR-induced DNA damage was the formation of micronuclei. The breakdown of the micronuclear envelope and subsequent exposure of self-DNA was associated with rapid cGAS translocation to the micronuclei and the onset of proinflammatory immune responses (121), a result that has since been corroborated in other studies (122, 158–163). Additionally, DNA damaging events often precipitate the release of mtDNA to the cytoplasm and this can similarly be recognized by cGAS (19, 164–169).

Importantly, mammalian cells appear to possess mechanisms that serve to limit cGAS activation to prevent excessive or prolonged activation that could lead to devastating autoimmunity. First, recent studies have determined that cGAS may primarily be localized to the nucleus in cells at rest (170, 171), rather than being cytosolic as was initially thought (95). In the nucleus, cGAS is tightly bound to nucleosomes where its DNA binding sites are prevented from interacting with nucleosomal DNA (172–177). Furthermore, while nuclear cGAS activation can occur, cGAMP production resulting from such activation is approximately 500-fold less than that generated when DNA is administered to the cytoplasm (171).

Second, cytoplasmic nucleases, such as three prime repair exonuclease 1 (TREX1) and deoxyribonuclease II (DNASE2) restrict cytoplasmic DNA accumulation thereby limiting cGAS activation (162). The impact of these nucleases is particularly apparent in inflammatory autoimmune disorders that occur due to mutations that result in their loss of function (141, 178, 179). Together, these studies reinforce the importance of the regulation of DNA sensing proteins, especially cGAS.

### 4.2 ZBP1

ZBP1 was the first cytosolic DNA sensor discovered (94) and its importance in host responses to viral infection have been extensively described (105, 180). ZBP1 was initially found to be a critical component in the detection of the dsDNA of HSV-1 (94) and other viruses in both peripheral and CNS cell types (105, 107, 108, 111, 181). More recently, it appears that this sensor may also recognize RNA motifs in addition to DNA (182). Furthermore, this cytosolic sensor has been implicated in the initiation of the necroptotic pathway of immunogenic cell death (105–108, 111, 182–188).

As shown in **Figure 2B**, upon binding to DNA, ZBP1 recruits RIPK3 that subsequently activates NF-κB to induce proinflammatory cytokine production (113). Additionally, ZBP1 has been shown to associate with TBK1 and IRF3 to regulate the activation of IRF3 and, thus, drive type-I IFN production (94). However, it is important to note that this receptor may act in a ligand and cell type specific manner, as ZBP1 knockdown was found to have no effect on exogenous DNA-induced IFN production in mouse embryonic fibroblasts (189) or a lung epithelial cell line (190), while similar knockdown significantly reduced B-DNA-induced IFN-β production in an immortalized murine fibroblast cell line (190).

Importantly, our current knowledge of the role of ZBP1 in responses to self-DNA remains limited, especially since the precise identity of the ligand(s) for ZBP1 remains controversial (181, 182, 187, 189, 191). However, there is data to support the notion that mtDNA serves as a ligand for ZBP1 (192, 193). A recent study has indicated that mtDNA release induced by low level oxidative stress, in the absence of detectable damage to nuclear DNA, elicits a type-I IFN response by pulmonary epithelial cells (192). Interestingly, in this study, fragments of mtDNA were shown to be released in exosomes that were capable of initiating further inflammatory responses in naïve epithelial cells (192). Furthermore, a second study reported the ability of glucose deprivation to induce mtDNA release *via* the actions of NOXA in FVB/NJ mice following implantation of the MVT-1 mammary cancer cell line (193), which subsequently initiated necroptosis in a ZBP1-dependent manner (193).

### 4.3 IFI16

IFI16 is a DNA sensor that is a member of the pyrin and HIN domain (PYHIN) family. While it was first identified in 1992 (194), it wasn’t until 2010 that it was characterized as a cytosolic DNA sensor capable of inducing IFN-β production in response to transfected exogenous DNA (96), and such responses have been reported to occur following direct interaction with STING (195) (**Figure 2C**). Interestingly, IFI16 exhibits both nuclear and cytosolic localization (196). In the nucleus, IFI16 has been shown to respond to viral DNA, which leads to the formation of an inflammasome that then translocates to the cytoplasm where it participates in the cleavage of pro IL-1β and IL-18 into their mature forms for release (114, 197, 198).

With regard to self-DNA detection, the murine ortholog of IFI16, IFI204, was found to mediate the detection of DNA released into the cytoplasm following DNA damage resulting from ataxia-telangiectasia mutated (ATM) deficiency in a murine model of ataxia telangiectasia (A-T) (123). Since the work of Mackenzie et. al (121), has indicated that cytosolic self-DNA recognition occurs due to the formation of micronuclei, it is possible that a similar mechanism underlies that ability of IFI204/IFI16 to perceive the presence of self-DNA, although further study will be necessary to confirm such a hypothesis.

While IFI16 is capable of binding self-DNA, it displays a preference for long non-self-DNA due to its ability to oligomerize into clusters, forming foci that are unable to bind nucleosomal self-DNA (199, 200). Alternatively, IFI16 may serve to detect DNA damage indirectly *via* the formation of a complex with DDR proteins that can subsequently initiate STING signaling (**Figure 2C**). Following etoposide-induced genotoxic stress, IFI16 has been found to combine with the DDR proteins ATM and poly(ADP-ribose) polymerase 1 (PARP1) to induce the formation of a STING signaling complex that results in the activation of NF-κB and proinflammatory cytokine production (200). This ATM-initiated nuclear mechanism results in far more rapid responses to DNA damage than those mediated by cGAS that require the formation and rupture of micronuclei (200). Furthermore, such responses predominantly result in the activation of NF-κB, rather than IRF3, resulting in a pro-inflammatory response (200). However, it is important to note that most of this work has been performed in keratinocytes and it remains to be determined whether such mechanisms exist in resident CNS cell type.

### 4.4 AIM2

AIM2 is another member of the PYHIN family that appears to have DNA sensing capabilities. First identified in 1997 (201), its role in dsDNA detection was not recognized until a decade later (97–99, 202). As shown in **Figure 2D**, AIM2 recruits the inflammasome adaptor protein ASC and procaspase-1 following binding to cytosolic DNA, forming an inflammasome complex that permits IL-1β and IL-18 maturation *via* the actions of caspase-1 (97–99, 202). While AIM2, like ZBP1, can also initiate immunogenic cell death pathways, AIM2 appears to initiate pyroptosis rather than necroptosis *via* the cleavage of gasdermin-D and subsequent pore formation (97–99, 202).

Importantly, AIM2 has been shown to detect cytosolic self-DNA and mtDNA (131, 203–205). The interaction between AIM2 and cytosolic self-DNA has primarily been studied in the context of autoimmune diseases that characteristically entail cytosolic DNA accumulation, such as arthritis, psoriasis, and systemic lupus erythematosus (131, 203, 206). Furthermore, pharmacological disruption of the nuclear envelope and the liberation of self-DNA to the cytosol has also been shown to induce AIM2 activation (205). As such, it has been inferred from these observations that AIM2 is capable of recognizing DNA and might be able to do so following micronuclei formation.

In addition, a limited number of studies suggest that AIM2 may also be localized to the nucleus in some cell types, such as macrophages (124), and can mediate responses to nuclear DNA damage (124, 207). For example, AIM2 has been reported to co-localize with the DNA damage marker gamma-H2A histone family member X (γH2AX) at sites of double strand breaks (DSBs) following IR exposure in macrophages, where it subsequently forms an AIM2-ASC-caspase-1 inflammasome complex to trigger pyroptotic cell death (124).

While the relative importance of AIM2-mediated DNA detection in the cytosol versus the nucleus remains unclear, it is noteworthy that the localization of the AIM2 inflammasome complex to sites of nuclear DNA DSBs occurs as rapidly as 8 hours following IR exposure (124). Furthermore, it is interesting that these inflammasome complexes have been observed to accumulate in the perinuclear region (124). However, it remains to be determined whether such accumulations involve additional interactions of AIM2 with cytosolic self-DNA.

## 5 Self-DNA detection in the CNS

To date, the mechanisms by which resident CNS cells can detect and respond to self- DNA are understudied, and it remain unclear whether the net result of such responses are beneficial or detrimental. Neuroinflammation, while crucial in protecting the brain against infection, can result in serious neurological damage if it is of inappropriate intensity or duration. Indeed, neuroinflammation that stems from DNA damage and/or deficient or defective DNA repair can underlie or exacerbate neurodegeneration in CNS disease states (11, 208–210). For example, the detection of cytosolic mtDNA and self-DNA by resident glial cells is a hallmark of CNS pathologies including AGS, AD, PD, A-T, and Huntington’s disease (HD) (44, 109, 160, 179, 211–216), as summarized in **Table 1**.

**Table 1:** Expression of DNA sensors by CNS cells and links to neurogenerative disorders.

Cell Type		Species	Sensor(s)	Stimuli/Model	Disease Association	References
Microglia	Primary	Human	cGAS-STING	Ganciclovir treatment	Not applicable	(217)
		Murine	cGAS-STING	ATM deficiency	Ataxia telangiectasia	(109)
				DNA transfection	Not applicable	(101)
				Tau protein aggregation	Alzheimer’s disease	(218)
			p204 (IFI16 ortholog)	DNA transfection	Not applicable	(101)
			AIM2	AIM2 deficient 5XFAD	Alzheimer’s disease	(219)
				MPTP-induced PD	Parkinson’s disease	(220)
				EAE	Multiple sclerosis	(221)
	Cell line	Human	cGAS-STING	DNA transfection	Not applicable	(100)
Astrocytes	Primary	Murine	p204 (IFI16 ortholog)	DNA transfection	Not applicable	(101)
			AIM2	EAE	Multiple sclerosis	(222)
Mixed Glia	Primary	Murine	cGAS-STING	α-synuclein preformed fibrils	Parkinson’s disease	(223)
Neurons	Primary	Human	cGAS-STING	Micronuclei formation	Huntington’s disease	(160)
			AIM2	DNA transfection	Not applicable	(224)
		Murine	cGAS-STING	Micronuclei formation	Huntington’s disease	(160)
			AIM2	DNA transfection	Not applicable	(224)
	Cell line	Murine	cGAS-STING	Cytosolic mtDNA accumulation	Amyotrophic lateral sclerosis	(225)
Whole brain	N/A	Murine	cGAS-STING	TREX deficiency	Aicardi-Goutières syndrome	(226)
				Rnaseh2a^G37S/G37S^ mutation	Aicardi-Goutières syndrome	(178)
				Cytosolic mtDNA accumulation	Amyotrophic lateral sclerosis	(225)
				Tau protein aggregation	Alzheimer’s disease	(218)
				APP/PS1	Alzheimer’s disease	(212, 227)
				EAE	Multiple sclerosis	(228)
			AIM2	APP/PS1	Alzheimer’s disease	(229, 230)
				ME7	Chronic neurodegeneration	(101)
			p204 (IFI16 ortholog)	ME7	Chronic neurodegeneration	(101)
		Zebrafish	IFI16	Cytosolic mtDNA	Parkinson’s disease	(231)

In some CNS disorders, such as A-T and AGS, the origins of cytosolic DNA accumulation are clear. In A-T, a critical kinase in DDRs, ATM, is defective and results in the accumulation of DSBs leading to the presence of cytosolic DNA (10, 123), while in AGS, mutations in the genes encoding products that process/degrade nucleic acids, such as TREX1 and RNAse H2, lead to cytosolic DNA accumulation and lethal autoimmunity in neonates (178, 226).

However, the origin of cytosolic DNA in other neurodegenerative diseases is either unclear or unknown. For example, in AD, amyloid-β (Aβ) plaques have been shown to induce oxidative stress that can cause mitochondrial dysfunction and subsequent mtDNA release into the cytosol (232). In contrast, the α-synuclein (α-Syn) fibrils that are commonly seen in PD can induce genomic DNA damage (223), while affected striatal neurons in HD patients and murine models of this disease show significantly higher numbers of micronuclei. However, the mechanisms underlying DNA damage in HD remain unknown (160).

We, and others, have shown that exogenous cytosolic DNA elicits reactive astrogliosis and microgliosis, and is associated with the production of proinflammatory and antiviral mediators by these cells (44, 100, 180, 212, 216, 233, 234). Importantly, the recent demonstration that glial cells express multiple cytosolic sensors has provided the means by which they perceive DNA. We showed that ZBP1 is expressed in microglia and astrocytes in an inducible manner following HSV-1 infection (106). In addition, Cox et. al (101), provided evidence that murine microglia and astrocytes express mRNA encoding cGAS, the p204 murine ortholog of IFI16, and AIM2 (101). Subsequently, we, and others, have extended these findings to demonstrate cGAS, IFI16, and AIM2, protein expression in human microglia and astrocytes, and have shown their ability to mediate glial immune responses to exogenous DNA administration (100, 102, 105, 224).

Transfection of exogenous DNA into human and murine primary glia and cell lines elicits the production of type-I IFNs and proinflammatory cytokines including CCL3, CCL5, CXCL2, TNF, IFN-β, and IL-6 (87, 88). The importance of the cytosolic DNA sensors, cGAS, IFI16 (and murine ortholog p204), and AIM2, in the generation of such responses is implied by the demonstration that dsDNA transfection into primary murine microglia and astrocytes significantly upregulates the expression of mRNA encoding these sensors (101). Importantly, we have shown that the responses of a human microglia cell line to intracellular exogenous DNA administration were significantly attenuated following CRISPR/Cas9 knockdown of cGAS expression (102). Furthermore, the secretion of mature IL-1β, downregulated dendritic growth, and enhanced axon extension of primary human and murine neurons elicited by dsDNA transfection was found to be dependent on the presence of AIM2 (224). As such, it is apparent that resident CNS cells are capable of responding to the presence of exogenous cytosolic DNA and do so *via* various sensor molecules.

With regard to the ability of resident CNS cells to perceive the presence of cytosolic self- DNA, glia have recently been shown to respond to mtDNA and self-DNA accumulation *via* sensors including cGAS and AIM2 (44, 109, 168, 214, 216, 223, 235, 236), as summarized in **Table 1**. For example, the addition of α-Syn preformed fibrils (PFF) to primary murine mixed glial cultures to simulate PD pathology resulted in DNA DSBs, an accumulation in cytosolic DNA, and subsequent activation of STING and TBK1 that resulted in type I IFN production (223). Importantly, these responses were attenuated by the pharmacological inhibition of STING activation (223). These findings are consistent with those in an *in vivo* mouse model where α-Syn-PFF similarly induced DNA damage, TBK1 activation, and IFN production by microglia *in situ* that preceded PD-like dopaminergic neurodegeneration, and the demonstration that substantia nigra pars compacta tissue from human PD patients show elevated STING protein levels that correlate with α-Syn-PFF accumulation (223).

The cGAS-STING axis has been implicated in the initiation of neurotoxic responses of primary microglial cultures to ATM mutations/deficiency that, again, results in the accumulation of cytosolic self-DNA and models A-T (109). This DNA sensing pathway has also been linked to striatal neuron cell death in HD (160). In primary human and murine HD-affected striatal neurons, Sharma et al. (160) reported a high incidence of micronuclei formation that coincided with cell death due to autophagy, and the increased expression of mRNA encoding CCL5 and CXCL10 that was abolished following cGAS depletion (160). Additionally, studies in mouse models of other neurodegenerative disorders have similarly indicated a role for cGAS in their progression. Most notably, models of AGS development that feature mutations of TREX1 and RNAse H2 have revealed that cGAS is essential for the initiation of the autoimmune responses associated with this disorder (178, 226).

Interestingly, AIM2-mediated responses have also been associated with neurodegenerative diseases but, in contrast to cGAS, such response appear to play a protective rather than a detrimental role, as summarized in **Table 1**. In primary murine microglia, AIM2 has been shown to alleviate the damaging neuroinflammation seen in the experimental autoimmune encephalitis (EAE) model of multiple sclerosis (MS), and other mouse models of AD and PD (219–221). In EAE, AIM2 was shown to negatively regulate DNA-PK activity in an inflammasome independent manner, and AIM2 deficiency was found to increase levels of microglial activation and peripheral leukocyte recruitment to the CNS (221). Furthermore, the inhibitory activity of AIM2 was found to be comparable to that of pharmacological DNA-PK inhibition (221). However, it should be noted that such findings conflict with other reports suggesting inflammasome components are required for the recruitment of peripheral T-cells to the CNS that exacerbate neuroinflammation in EAE (237, 238). As such, the role of AIM2 in inflammatory CNS diseases appears complex as it relates to both resident glia and peripheral leukocytes and further research is clearly required.

In the 5XFAD model of AD, deletion of AIM2 resulted in a decrease in Aβ deposition, but caused an elevation in the production of the key inflammatory cytokines IL-6 and IL-18, further supporting a negative regulatory role for AIM2 in neuroinflammation (219). Finally, in a neurotoxin (N-methyl-4-phenyl-1, 2, 3, 6-tetrahydropyridine, MPTP)-induced PD mouse model, AIM2 activation served to limit cGAS activity *via* interference with protein kinase B (AKT)-mediated IRF3 phosphorylation, and conditional knockout of AIM2 in microglia, but not peripheral cells, exacerbated PD-like disease severity (220).

As such, while it is clear that cytosolic DNA sensors play critical roles in the initiation and/or progression of CNS pathologies in animal models (as summarized in **Table 1**), their specific roles can often appear contradictory and so may be sensor and/or disease condition specific. Additionally, while light has been shed on the beneficial/detrimental effects mediated by these receptors in murine CNS cells and neurodegenerative disorder models, it remains unclear whether cytosolic DNA sensors exert similar functions in human disease. A better understanding of these DNA sensors in the context of human neurons/glia and patients with CNS disorders could identify new targets for therapeutic intervention to limit neuroinflammation and/or to promote beneficial immune responses.

## 6 Clinical implications and concluding remarks

Taken together, it has become apparent that resident CNS cells play a critical role in the protective and detrimental immune responses associated with CNS infection and damage, and the development/progression of neurological disorders. Furthermore, it is increasingly clear that such responses are initiated *via* the detection of DAMP and PAMP motifs that are associated with cellular damage and infectious agents, respectively. The principal glial cells, microglia and astrocytes, express various cell-surface, endosomal, and cytosolic members of multiple PRR families that trigger immune mediator production to foster neuroinflammation and recruit leukocytes to the CNS. Importantly, glia can constitutively, and/or be induced to, express PRRs that detect the presence of DNA in the cytosolic compartment. While these sensors were initially characterized as components in the detection of viral and bacterial nucleic acids by microglia and astrocytes, it is now recognized that molecules, including cGAS, IFI16, and AIM2, can play important roles in the generation of responses to the presence of self-DNA in the cytosol resulting from DNA damaging insults, such as IR or oxidative stress, or deficient/defective DNA repair. As such, these mechanisms might be targetable to either augment glia-mediated responses that serve to protect against tumorigenesis, or prevent the inflammatory responses of these cells that initiate or exacerbate damaging neuroinflammation.

To date, several small molecule inhibitors and agonists have been developed for cGAS (239) and AIM2 (240), as well as for the downstream adaptor proteins STING (241–245) and RIPK1/3 (246). These agents have shown good efficacy in both peripheral cells and isolated glia where pharmacological inhibition of these components has led to significant reductions in the production of immune mediators including IFN-β, IL-1β, and IL-6, following activation (105, 240, 241, 245, 247–250). Indeed, the STING agonists, TAK-676 (NCT04420884, NCT04879849) and E7766 (NCT04144140), are currently being tested clinically for the treatment of advanced or metastatic solid tumors, lymphomas, and leukemias, as adjunctive agents to conventional chemotherapy. As such, it might be possible in the future to target the DNA sensors or their downstream adaptor proteins associated with CNS cancers and neurodegenerative diseases.

However, considerable hurdles remain for the development and use of such agents as the available data regarding the specific role of each cytosolic DNA sensor in CNS disorders can be unclear or even contradictory. This may be due to species-specific differences, such as in the ability of STING to be activated by cGAMP (251), or it may be that the glial responses initiated by the presence of cytosolic DNA are sensor, cell type, or even disease stage, dependent. In addition, the ability of many small molecule inhibitors and agonists that target cytosolic DNA sensors to cross the BBB and their efficacy in the brain have not been determined. Indeed, marked differences in the effectiveness of such agents has been noted between studies in isolated glia and those in murine model or clinical settings, primarily due to poor BBB penetration, as reviewed elsewhere (252). While various methods have been successfully employed to circumvent this issue, including BBB disruption and intracerebral, intrathecal, or intranasal, delivery, each carries its own problems, such as neurotoxicity (BBB disruption) or a high degree of invasiveness (intrathecal delivery) (252). Finally, the potential for adverse side-effects of agents targeting DNA sensor mechanisms remains unclear, as no DNA sensor inhibitors or agonists are currently undergoing clinical trials for their efficacy in the treatment of CNS tumors or neurodegenerative disease.

Clearly, more study of these novel cytosolic DNA sensor pathways is warranted given our current lack of understanding of the role of each in glial functions in the context of specific CNS disorders and their stages. Furthermore, successfully establishing the *in situ* efficacy of agonists/antagonists of these DNA sensor pathways in the CNS, and solving the issue of delivery across the BBB, could represent an exciting new therapeutic modality that might be used alone or in conjunction with existing approaches to improve the treatment of a wide range of CNS pathologies.

## Author contributions

AS and IM co-wrote this literature review article. All authors contributed to the article and approved the submitted version.
